# Virological findings from the SARA trial: boosted PI monotherapy as maintenance second-line ART in Africa

**DOI:** 10.1186/1758-2652-13-S4-O20

**Published:** 2010-11-08

**Authors:** D Pillay, R Goodall, CF Gilks, D Yirrell, D Gibb, M Spyer, P Kaleebu, P Munderi, C Kityo, A McCormick, J Nkalubo, F Lyagoba, M Chirara, J Hakim, DART

**Affiliations:** 1 University College London, London, UK; 2 MRC Clinical Trials Unit, London, UK; 3 Imperial College, London, UK; 4 Ninewells Hospital and Medical School, Dundee, UK; 5 MRC/UVRI Uganda Research Unit on AIDS, Entebbe, Uganda; 6 Joint Clinical Research Centre, Kampala, Uganda; 7 University of Zimbabwe, Harare, Zimbabwe; 8 Virology Group, and Trial Team

## Background

The SARA trial recently demonstrated a non-inferior CD4 response, over median follow-up of 60 weeks, with a boosted protease inhibitor monotherapy (bPImono) maintenance second-line regimen compared with continuous combination therapy (CT), suggesting this approach could maintain effectiveness whilst improving tolerability and decreasing costs [International AIDS Conference 2010, LBPE16]. Analysis of virological response and genotypic drug resistance is reported here.

## Methods

Eligible participants in the DART trial who received 24 weeks of lopinavir/ritonavir-containing second-line CT were randomised to maintain current CT or to reduce to bPImono within a nested pilot trial (SARA). No real-time virology was performed, but stored plasma samples from time at switch to second-line, randomisation after 24 weeks of second-line, and 24 weeks after randomisation were assayed for HIV-1 RNA viral load (VL) by Roche Amplicor v1.5. Genotypic resistance was assessed on samples with VL >1000 c/ml at this latest time point, along with paired samples at switch to second-line. All analyses are intention-to-treat.

## Results

192 participants were randomised to CT (n=95) or bPImono (n=97). 77% (135/173) had VL<50 c/ml at randomisation. 44 (23%) participants were taking bPI with NRTI only, 29 (15%) with NNRTI only, and 119 (62%) with both. Virological suppression at week 24 was higher (trend test p=0.007) for participants on CT vs bPImono: 77% (70/91) vs 60% (56/94) had VL <50 c/ml, 90% (82) vs 74% (72) had VL <200 c/ml, and 94% (86) vs 84% (81) had VL <1000 c/ml. Restricting to patients with VL <50 c/ml at randomisation, 85% (57/67) vs 66% (43/65) had VL <50 c/ml at week 24. Of the 18 participants with VL >1000 c/ml at week 24, 12 (2 CT, 10 bPImono) have been assessed genotypically. IAS major PI mutations at week 24, not present at switch to second-line, were detected in 2 bPImono participants only. One participant (VL=3600 c/ml) had I54V only, the other (VL=1490 c/ml) M46IM+V82AV. Both isolates were considered fully susceptible to darunavir.

## Conclusions

In this study based on retrospective virological testing, bPImono following 24 week second-line induction was associated with an increase in low level viraemia, although generally in the absence of PI resistance. Longer-term trials are required before definitive conclusions can be drawn about the effectiveness of PI monotherapy in populations without access to virological monitoring.

